# Myeloperoxidase-Associated Membranous Nephropathy in Antineutrophil Cytoplasmic Antibody-Associated Glomerulonephritis

**DOI:** 10.1016/j.ekir.2024.04.041

**Published:** 2024-04-23

**Authors:** Kenta Tominaga, Etsuko Toda, Kazuhiro Takeuchi, Shoichiro Takakuma, Emi Sakamoto, Hideaki Kuno, Yusuke Kajimoto, Yasuhiro Terasaki, Shinobu Kunugi, Mika Terasaki, Hiroyasu Goto, Toshihiko Imakiire, Naoki Oshima, Akira Shimizu

**Affiliations:** 1 Department of Analytic Human Pathology, Nippon Medical School, Tokyo, Japan; 2 Division of Nephrology and Hypertension, The Jikei University School of Medicine, Tokyo, Japan; 3 Division of Pathology, Nippon Medical School Hospital, Tokyo, Japan; 4 Department of Nephrology and Endocrinology, National Defense Medical College, Saitama, Japan

**Keywords:** antigen, antineutrophil cytoplasmic antibody, glomerular basement membrane, glomerulonephritis, membranous nephropathy, myeloperoxidase

## Abstract

**Introduction:**

Antineutrophil cytoplasmic antibody (ANCA)-associated glomerulonephritis (GN) is characterized by pauci-immune crescentic GN. Myeloperoxidase ANCA-associated GN (MPO-ANCA GN) with membranous nephropathy (MN), where bright granular capillary MPO and IgG staining along the glomerular basement membrane (GBM) is present, has been reported; however, its clinicopathological features remain unclear.

**Methods:**

We investigated 7 MPO-ANCA GN with MN and 11 control cases (6 MPO-ANCA GN and 5 primary MN cases). Proteomics of laser microdissected glomeruli followed by immunohistochemical analysis was performed to identify causal antigens in MPO-ANCA GN with MN. We described the clinicopathological features of MPO-associated MN compared with those of MPO-ANCA GN and primary MN.

**Results:**

We detected proteomic MPO and granular capillary MPO deposits in all MPO-ANCA GN with MN cases. Confocal microscopy revealed MPO and IgG colocalization along the GBM. MPO-associated MN clinicopathological features include greater proteinuria, a higher fibrous crescent rate, and a lower MPO-ANCA titer than MPO-ANCA GN. The estimated glomerular filtration rate (eGFR) and urinary protein excretion were lower in MPO-associated MN than in primary MN.

**Conclusion:**

MPO-associated MN, a unique type of secondary MN where MPO serves as the causal antigen, is a subset of MPO-ANCA GN with MN. Prolonged periods of MPO-ANCA GN and a low MPO-ANCA titer might be related to MPO-associated MN development.

## Graphical abstract

**Figure:**
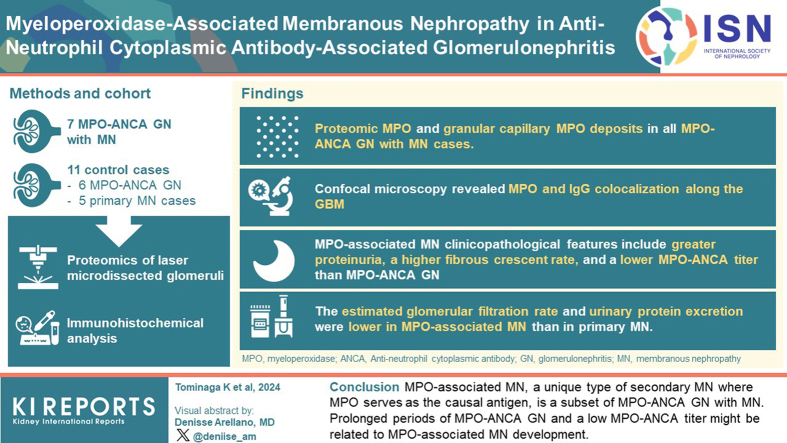

ANCA GN is characterized as a pauci-immune type of crescentic GN.^1^ However, several studies have demonstrated the presence of MN as a rare concomitant pattern of ANCA GN.^2^, ^3^, ^4^ A granular pattern of MPO and IgG deposition along the GBM was also observed in patients with MPO-ANCA GN and MN.^5^, ^6^, ^7^ Furthermore, MPO can be detected within subepithelial electron-dense deposits (EDD) in patients with MPO-ANCA GN accompanied by MN lesions.^8^ Although MPO-ANCA GN and MN might be closely related, the clinicopathological characteristics and underlying MPO-ANCA GN with MN development are unknown. Therefore, we performed proteomic analysis followed by MPO staining, employing immunohistochemistry (IHC) and immunofluorescence (IF) and assessed the localization of MPO and IgG using confocal microscopy in patients with MPO-ANCA GN with MN. Moreover, we described the clinicopathological characteristics of MPO-associated MN compared with MPO-ANCA GN and primary MN.

## Methods

### Patients

**Figure 1:**
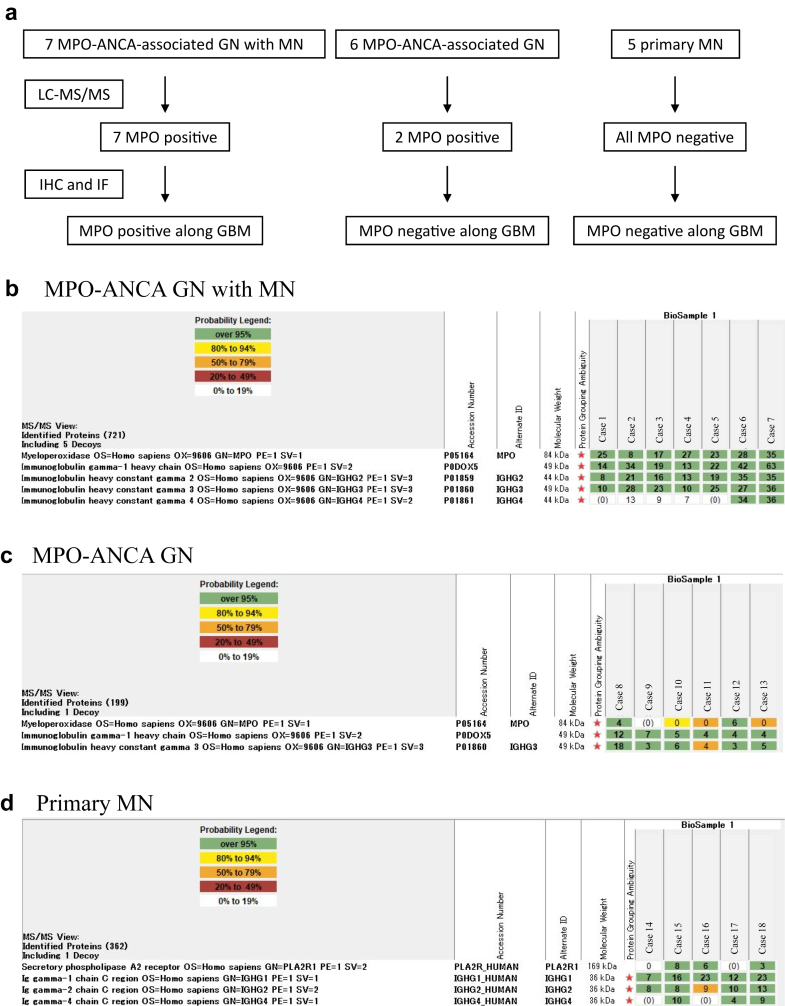
Flowchart of all investigated cases and proteomic detection of myeloperoxidase (MPO) in MPO antineutrophil cytoplasmic antibody (ANCA)-associated glomerulonephritis (MPO-ANCA GN) with membranous nephropathy (MN) and MPO-ANCA GN. (a) We performed liquid chromatography with tandem mass spectrometry (LC-MS/MS) on all cases, including 7, 6, and 5 cases of MPO-ANCA GN with MN, MPO-ANCA GN, and primary MN, respectively. MPO was detected in all cases of MPO-ANCA GN with MN and 2 cases of MPO-ANCA GN. Subsequently, we performed IHC and IF staining for MPO on all cases, of which 7 MPO-ANCA GN with MN cases showed glomerular capillary positivity for MPO on IHC and IF staining. (b) Moderate to high spectral counts of the 7 MPO-ANCA GN with MN cases. Numbers in green boxes represent spectral counts of LC-MS/MS matched to a respective protein. The top panel shows the MPO spectral counts. The second to fifth panels show IgG1, IgG2, IgG3, and IgG4, respectively. (c) Low spectral counts in 2 MPO-ANCA GN cases. Numbers in green, yellow, and orange boxes represent spectral counts of LC-MS/MS matched to a respective protein. The top panel shows the MPO spectral counts. The second panel shows IgG1, and the bottom panel shows IgG3. (d) Low spectral counts in 3 primary MN cases. Numbers in green and orange boxes represent spectral counts of LC-MS/MS matched to a respective protein. The top panel shows the phospholipase A2 receptor spectral counts, whereas the second to fourth panels show IgG1, IgG2, and IgG4, respectively.

We selected 18 patients with MPO-ANCA GN with MN, MPO-ANCA GN, and primary MN in the Department of Nephrology and Endocrinology of the National Defense Medical College from January 2011 to January 2023 (Figure 1a). Among 49 cases of MPO-ANCA positive crescentic GN, 7 were MPO-ANCA GN with MN cases. There were 6 MPO-ANCA GN cases with sufficient glomeruli and without deposition of immune complexes, complement, or EDD. Here, 5 cases of primary MN, which was defined as MN with serological antiphospholipase A2 receptor antibody positivity and glomerular phospholipase A2 receptor positivity along the GBM, were present; this also ruled out secondary MN due to malignancy, autoimmune diseases, infections, and medications. The clinical and laboratory information of all cases was obtained from digital medical records. Liquid chromatography with tandem mass spectrometry (LC-MS/MS) was performed on all cases. After that, IHC and IF staining were performed to confirm the LC-MS/MS results. This study protocol was conducted in accordance with the principle of the Declaration of Helsinki and was approved by the Research Ethics Committee of Nippon Medical School, Tokyo, Japan (approval number: M-2022-087). Informed consent was obtained from the websites of Nippon Medical School and National Defense Medical College.

### Mass Spectrometry Proteomic Analysis Using Laser Microdissection and LC-MS/MS

For each case, 10 μm thick formalin-fixed, paraffin-embedded tissues were obtained, mounted on a special polyethylene naphthalate membrane laser microdissection slide, and dissected using a Leica dissector (Leica LMD6000, Leica Microsystems, Mannheim, Germany). The microdissected tissue samples ranged from 400,000 to 1200,000 μm^2^ per case. These fragments were collected and digested with tryptic peptides. The tryptic digestion of the samples was identified by LC-MS/MS using amazon ETD (Bruker Daltonics, Billerica, MA). All LC-MS/MS data were analyzed using Mascot and X! Tandem and searched using the Swiss Prot human database. LC-MS/MS–based peptide and protein identifications were validated using Scaffold (Proteome Software Inc., Portland, OR). Peptide identification was accepted at >95% probability with a 2 peptide minimum as specified by the peptide algorithm.

### IHC Staining for MPO

Formalin-fixed, paraffin-embedded tissues were sectioned at 4 μm, and IHC staining was conducted. Briefly, the paraffin slides were placed in Histofine buffer (Nichirei, Tokyo, Japan) at pH 9 and heated to 120 °C for 20 minutes. The primary and secondary antibodies for MPO staining were monoclonal rabbit antihuman MPO (#718261; Nichirei, Tokyo, Japan) and Histofine Simple Stain MAX PO (MULTI; Nichirei, Tokyo, Japan), respectively. Finally, the slides were visualized using 3, 3′-diaminobenzidine tetrahydrochloride.

### IF Staining for MPO and Colocalization Analysis

IF staining was performed on the formalin-fixed, paraffin-embedded sections of all cases using monoclonal rabbit antihuman MPO (Nichirei, Tokyo, Japan) and secondary antibody Alexa Fluor 555 conjugated donkey antirabbit IgG (Thermo Fisher Scientific, MA).

First, we examined the colocalization of MPO and IgG along the GBM through confocal microscopy using ZEISS LSM 980 and analyzed them using ZEN Microscopy Software (version 3.8; ZEN, Carl Zeiss, Germany). The formalin-fixed, paraffin-embedded sections of 7 MPO-associated GN with MN cases were incubated with a combination of monoclonal rabbit antihuman MPO and antihuman IgG goat polyclonal antibody (Zymed, San Francisco, CA). Next, the samples were incubated with a combination of secondary antibody Alexa Fluor 555 conjugated donkey antirabbit IgG and secondary antibody donkey Alexa Fluor 488 conjugated antigoat IgG (Thermo Fisher Scientific, MA).

### Statistical Analyses

Statistical analyses were performed using GraphPad Prism 9 (GraphPad Software Inc., San Diego, CA). Continuous data are presented as median (interquartile range). The Mann-Whitney *U* test was used to assess group differences. Statistical significance was set at *P* < 0.05.

## Results

### LC-MS/MS Detection of MPO in MPO-ANCA GN With MN

Glomerular dissection was performed using a Leica dissector with a range of 400,000 to 1,000,000 μm^2^ per case, and MPO was detected in 7 MPO-ANCA GN with MN cases (Figure 1b). The mean total spectral count of MPO was 23.3 (SD, ± 8.7) per MPO-ANCA GN with MN case. Conversely, the mean total spectral count of MPO was 1.7 (SD, ± 2.7) and 0 (SD, ± 0) in MPO-ANCA GN (Figure 1c) and primary MN (Figure 1d) cases, respectively. All MPO-ANCA GN with MN cases showed no spectral counts for phospholipase A2 receptor or neural epidermal growth factor like 1. All 4 IgG subclasses were identified in MPO-associated MN as follows: IgG1 was the most representative Ig (mean ± SD: 29.6 ± 18.2), followed by IgG3 (22.7 ± 9.6), IgG2 (21.0 ± 10.4), and IgG4 (14.1 ± 15.0).

### Glomerular Capillary Staining for MPO in all MPO-ANCA GN With MN Cases

**Figure 2:**
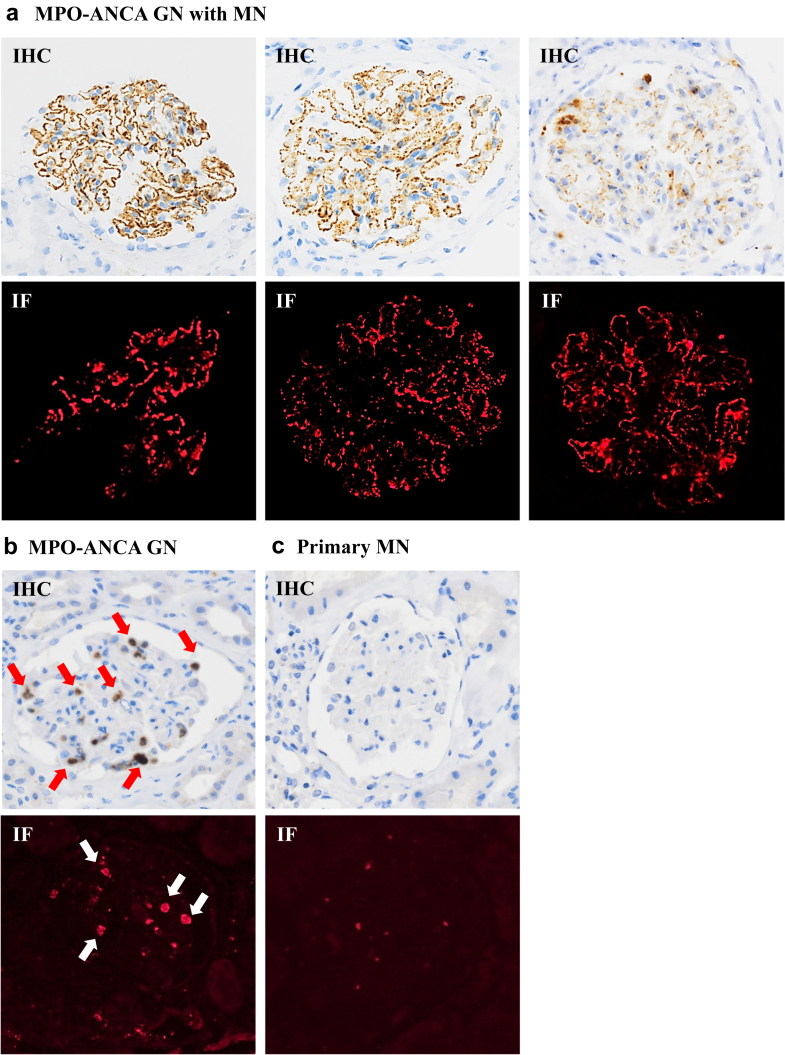
Immunohistochemistry (IHC) and immunofluorescence (IF) staining for myeloperoxidase (MPO) in MPO antineutrophil cytoplasmic antibody (ANCA)-associated glomerulonephritis (MPO-ANCA GN) with membranous nephropathy (MN), MPO-ANCA GN, and primary MN. (a) Fine granular staining for MPO along the glomerular basement membrane (GBM) was observed in MPO-ANCA GN with MN. (b and c) A lot of neutrophil staining for MPO is found in MPO-ANCA GN (red and white arrows), whereas no glomerular capillary staining for MPO is observed in MPO-ANCA GN and primary MN on IHC and IF staining.

IHC staining for MPO was performed in all cases. All 7 MPO-ANCA GN with MN cases showed fine granular staining for MPO along the GBM. Segmental granular staining for MPO along the GBM was also observed in cases 3 and 5. Although neutrophils showed MPO-positive staining, no MPO staining was observed along the Bowman’s capsule, tubular basement, or capillary vessel. However, all control cases, including MPO-ANCA GN and primary MN, were negative for MPO along the GBM. Intraglomerular neutrophil infiltration (red and white arrows, Figure 2) was significant in 2 patients with MPO-ANCA GN (cases 8 and 12), who showed low spectral counts for MPO on LC-MS/MS.

IF staining for MPO was performed in 7 MPO-ANCA GN with MN cases to reveal MPO localization. All 7 cases showed bright granular staining for MPO along the GBM (red, Figure 2). Similar to the IHC results, segmental granular staining for MPO along the GBM was observed in cases 3 and 5. Moreover, electron microscopy demonstrated segmental subepithelial deposits in all 7 cases (Supplementary Figure S1). All control cases were negative for MPO along the GBM. Figure 2 illustrates representative IHC and IF staining for MPO, and Table 1 presents all IHC and IF staining results for MPO.

**Table 1:** Immunohistochemistry and immunofluorescence results in all cases

a. Immunohistochemistry and immunofluorescence results in MPO-associated MN cases							
	Case 1	Case 2	Case 3	Case 4	Case 5	Case 6	Case 7
Capillary MPO (IHC)	global	global	seg	global	seg	global	global
Capillary MPO (IF)	global	global	seg	global	seg	global	global
Neutrophil infiltration	2+	1+	2+	2+	2+	2+	2+

b. Immunohistochemistry and immunofluorescence results in MPO-ANCA GN cases						
	Case 8	Case 9	Case 10	Case 11	Case 12	Case 13
Capillary MPO (IHC)	−	−	−	−	−	−
Capillary MPO (IF)	−	−	−	−	−	−
Neutrophil infiltration	3+	1+	1+	2+	3+	2+

c. Immunohistochemistry and immunofluorescence results in Primary MN cases					
	Case 14	Case 15	Case 16	Case 17	Case 18
Capillary MPO (IHC)	−	−	−	−	−
Capillary MPO (IF)	−	−	−	−	−
Neutrophil infiltration	1+	1+	1+	1+	1+

−, negative; 1+, few neutrophils (< 3 neutrophils/glomeruli); 2+, moderate neutrophils (3–9 neutrophils/glomeruli); 3+, massive neutrophils (> 10 neutrophils/glomeruli); ANCA, antineutrophil cytoplasmic antibody; global, global MPO positive (> 50% in capillary); IF, immunofluorescence; IHC, immunohistochemistry; MN, membranous nephropathy; MPO, myeloperoxidase; MPO-ANCA GN, MPO-ANCA-associated glomerulonephritis; seg, segmental MPO positive (< 50% in capillary).

### Colocalization of MPO and IgG in MPO-ANCA GN With MN Cases

**Figure 3:**
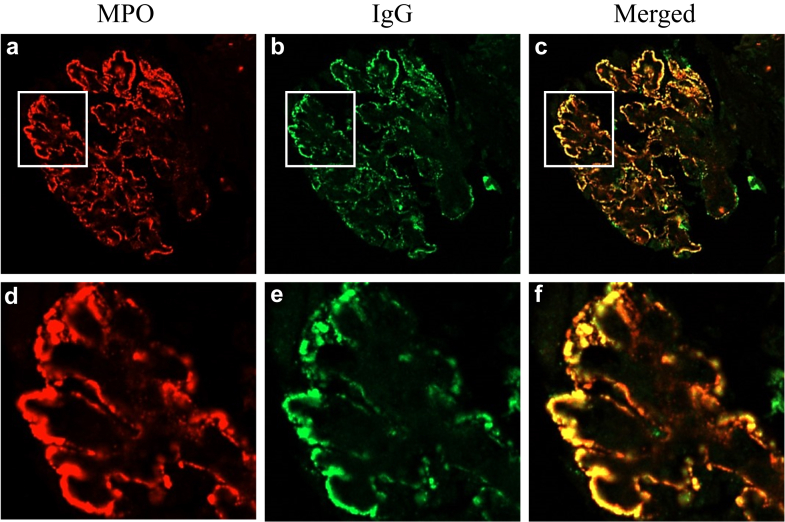

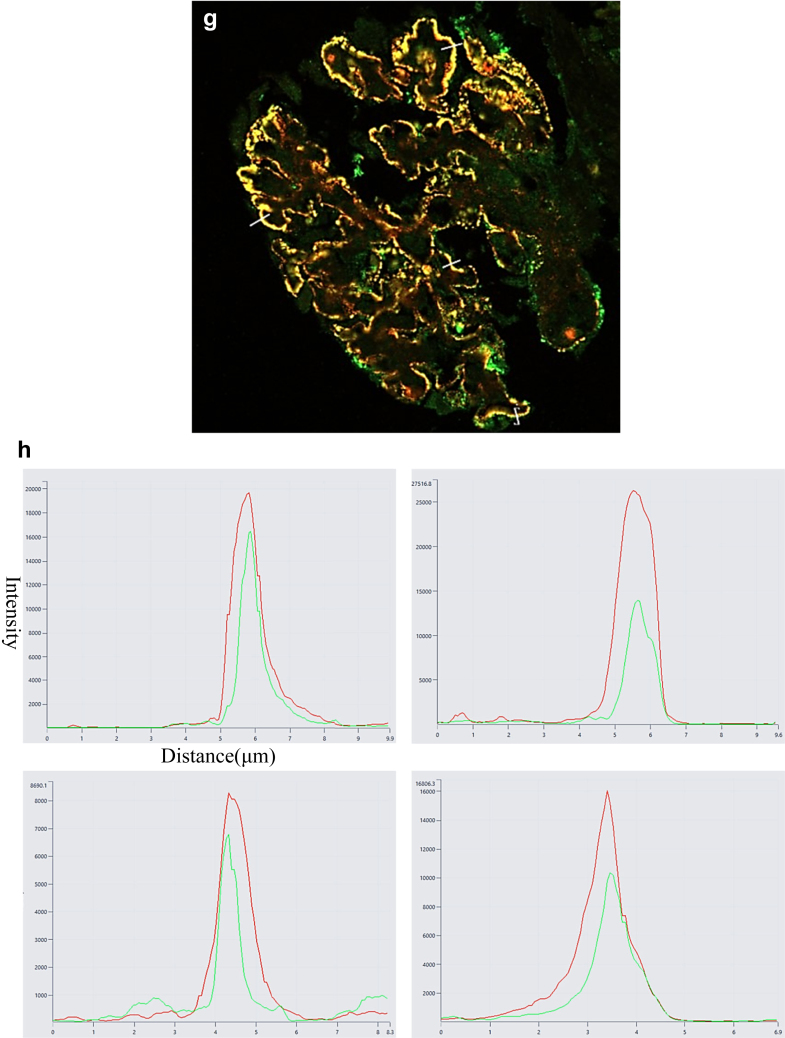
Identification of myeloperoxidase (MPO) and IgG in glomerular immune complexes in MPO-associated membranous nephropathy (MN) cases using confocal immunofluorescence microscopy. Glomerular double labeling with (a and d) anti-MPO (red) and (b and e) antihuman IgG (green) is shown, as well as the merged images (c and f) with anti-MPO and antihuman IgG (yellow). (g) The whole image of the merged image. The white lines are located across the sections of representative glomerular capillary walls to record the fluorescence. (h) Quantitative analysis of fluorescence on the white lines. The superimposition and same peak of the 2 signals suggest that the capillary immune complexes comprise MPO (red) and IgG (green).

We performed confocal IF to reveal whether MPO and IgG colocalized in MPO-ANCA GN with MN cases because IgG deposition is generally observed with the causative antigen in primary MN. Bright granular staining for MPO (red) and IgG (green) was revealed along the GBM (Figure 3a, b, d, and e). However, an overlap between MPO and IgG showed a yellow signal (Figure 3c, f, and g). Quantitative laser analysis showed that MPO and IgG colocalized in the GBM (Figure 3h).

### Clinicopathological Characteristics of MPO-Associated MN

We detected 7 MPO-associated MN cases: 5 and 2 cases involved females and males, respectively. The patients’ median age was 63.0 (56.0–68.5) years. In addition, the median eGFR at baseline and 12 months postrenal biopsy was 13.9 (11.1–38.1) and 28.7 (17.6–49.6) ml/min per 1.73 m^2^, respectively. At baseline and 12 months postrenal biopsy, the median urinary protein excretion was 3.0 (2.5–5.4) and 1.3 (0.5–1.5) g/g Cr, respectively. The median ANCA titer for MPO was 25.6 (20.6–72.4) U/ml. In 2 patients with MPO-associated MN, MPO-ANCA titers were positive for >10 years before renal biopsy (Supplementary Table S1). Remission induction treatment did not differ between MPO-associated MN and MPO-ANCA GN cases. In Table 2, we present all clinical data of MPO-associated MN cases; however, antiphospholipase A2 receptor antibodies were not investigated in all MPO-associated MN cases. In Table 3, Table 4, we present the clinical data of the cases of MPO-ANCA GN and primary MN as controls for MPO-associated MN.

**Table 2:** Clinical data of MPO-associated MN

Case	Age, yr	Sex	eGFR (ml/min per 1.73 m^2^)	eGFR after 12 mo (ml/min per 1.73 m^2^)	U-Pro (g/gCr)	U-Pro after 12 mo (g/gCr)	MPO-ANCA (U/ml)	Remission induction therapy	Additional therapy
1	63	F	61.6	51.3	2.65	0.13	25.6	PSL	None
2	77	F	13.9	16	3.91	1.81	21	PSL	None
3	64	F	9.9	9.5	2.35	1.29	64.3	mPSL 0.5 g × 3 days, PSL	None
4	73	M	6.9	19.1	3.04	0.28	20.1	mPSL 0.5 g × 3 days, PSL	None
5	60	F	12.3	28.7	1.58	0.68	80.5	mPSL 0.5 g × 3 days, PSL	CY, AZA
6	34	F	46	54.7	6.8	1.26	245	mPSL 0.5 g × 3 days, PSL	MMF
7	52	M	30.1	47.8	9.42	1.74	19.4	PSL	MZB, AZA

M, male; F, female; ANCA, antineutrophil cytoplasmic antibody; AZA, azathioprine; CY, cyclophosphamide; eGFR, estimated glomerular filtration rate at the time of renal biopsy; F, female; M, male; MMF, mycophenolate mofetil; MN, membranous nephropathy; MPO, myeloperoxidase; mPSL, methylprednisolone; MZB, mizoribine; PSL, prednisolone; U-Pro, urinary protein creatinine ratio.

**Table 3:** Clinical data of MPO-ANCA GN

Case	Age, yr	Sex	eGFR (ml/min per 1.73 m^2^)	eGFR after 12 mo (ml/min per 1.73 m^2^)	U-Pro (g/gCr)	U-Pro after 12 mo (g/gCr)	MPO-ANCA (U/ml)	Remission induction therapy	Additional therapy
8	67	M	29.6	41.3	1.78	0.12	201	mPSL 0.5 g × 3 days, PSL	None
9	80	F	15.1	29.2	0.32	0.1	85.8	mPSL 0.5 g × 3 days, PSL	None
10	73	F	37	33.2	1.02	0.18	34.7	PSL	MZB
11	71	F	5	37.1	0.21	0.14	367	mPSL 0.5 g × 3 days, PSL	MZB
12	62	M	4.5	13.4	1.83	0.42	107	mPSL 0.5 g × 3 days, PSL	None
13	73	M	27.3	34.9	4.2	0.47	343	PSL	None

M, male; F, female; ANCA, antineutrophil cytoplasmic antibody; eGFR, estimated glomerular filtration rate at the time of renal biopsy; GN, glomerulonephritis; MPO, myeloperoxidase; mPSL, methylprednisolone; MZB, mizoribine; PSL, prednisolone; U-Pro, urinary protein creatinine ratio.

**Table 4:** Clinical data of primary MN

Case	Age, yr	Sex	eGFR (ml/min per 1.73 m^2^)	eGFR after 12 mo (ml/min per 1.73 m^2^)	U-Pro (g/gCr)	U-Pro after 12 mo (g/gCr)	Anti-PLA2R antibody (index value)	Remission induction therapy	Additional therapy
14	64	F	78.9	66	19.3	2.57	5.1 (+)	mPSL 0.5 g × 3 days, PSL	None
15	74	M	59.9	47.3	4.53	0.56	1.6 (+)	mPSL 0.5 g × 3 days, PSL	None
16	83	F	30.8	48.6	16.7	1.04	4.6 (+)	PSL	None
17	57	F	74.5	55.5	6.06	1.77	4.8 (+)	mPSL 0.5 g × 3 days, PSL	MZB
18	74	M	46.6	29.2	6.83	0.63	3.4 (+)	mPSL 0.5 g × 3 days, PSL	None

M, male; F, female; eGFR, estimated glomerular filtration rate at the time of renal biopsy; MN, membranous nephropathy; mPSL; methylprednisolone, MZB, mizoribine; PLA2R; phospholipase A2 receptor; PSL, prednisolone; U-Pro, urinary protein creatinine ratio.

The pathological features of 7 MPO-associated MN cases showed crescentic GN with subepithelial EDD followed by fibrous crescent and glomerular IgG and C3 deposition. Overall, a mean of 26.0 ± 7.4 glomeruli were evaluated, of which 47.6% ± 18.5%, 31.3% ± 15.3%, and 16.3% ± 7.3% formed crescents, cellular or fibro cellular crescents, and fibrous crescents, respectively. Fibrinoid necrosis, as demonstrated using capillary IgG (1–2+/2) and C3 (1–2+/2) via IF staining, was noted in 2 of the 7 cases. Among the 7 cases, 4 and 1 showed positive staining for IgM (± −1+/2) and IgA (1+/2) and complement component 1q (C1q) (±/2), respectively. However, tubuloreticular inclusions were not observed. In Table 5, we present the pathological features of the patients.

**Table 5:** Pathological features of MPO-associated MN

Case	Glomeruli	Crescents (%)	Cellular or Fibrocellular crescents (%)	Fibrous crescents (%)	Fibrinoid necrosis	Immunofluorescence	Electron dense deposits
1	19	10.5	5.3	5.3	Not present	IgG (1+), C3 (1+)	stage III
2	35	54.3	31.4	22.9	Not present	IgG (1+), IgM (1+), C3 (1+)	stage II
3	22	50.0	36.4	13.6	Not present	IgG (1+), C3 (1+)	stage I
4	35	37.1	17.1	20.0	Present	IgG (2+), C3 (2+)	stage II
5	26	57.7	42.3	15.4	Present	IgG (1+), IgM (1+), C3 (1+)	stage II
6	19	63.2	36.8	26.3	Not present	IgG (2+), IgA (1+), IgM (1+), C3 (2+)	stage I
7	28	60.7	50.0	10.7	Not present	IgG (1+), IgM (±), C3 (2+), C1q (±)	stage II

C1q, complement component 1q; IgA, immunoglobulin A; IgG, immunoglobulin G; IgM, Immunoglobulin M; MN, membranous nephropathy; MPO, myeloperoxidase.

Immunofluorescence microscopy (−, ±, 1+, 2+ represents intensity of scoring; total score of 4).

Electron-dense deposits (I–Ⅳ represents Ehrenreich-Churg stages of membranous nephropathy).

### Comparison MPO-Associated MN Cases With MPO-ANCA GN and Primary MN Cases

We detected 7 MPO-associated MN cases based on IHC and IF staining for MPO and compared the clinicopathological characteristics of MPO-associated MN cases with those of MPO-ANCA GN and primary MN cases (Table 6). Patients with MPO-associated MN showed a significantly higher urinary protein level after 12 months (1.3 [0.5–1.5] vs. 0.2 [0.1–0.4] g/gCr; *P* = 0.035), a higher rate of fibrous crescents (15.4 [12.2–21.4] vs. 0.0 [0.0–1.8]%; *P* = 0.012), and a lower MPO-ANCA titer (25.6 [20.6–72.4] vs. 154.0 [91.1–307.5] U/ml; *P* = 0.035) than those with MPO-ANCA GN. The other laboratory data were not statistically significant. Meanwhile, eGFR (13.9 [11.1–38.1] vs. 59.9 [46.6–74.5] ml/min per 1.73 m^2^; *P* = 0.0303) and urinary protein excretion (3.0 [2.5–5.4] vs. 6.8 [6.1–16.7] g/gCr; *P* = 0.048) were lower in patients with MPO-associated MN than in those with primary MN. No other significant differences were observed between the groups.

**Table. 6:** Clinical and pathological features of MPO-associated MN compared with those of MPO-ANCA GN and primary MN

Clinical and pathological data	MPO-associated MN	MPO-ANCA GN	Primary MN	*P* value (MPO-associated MN vs. MPO-ANCA GN)	*P* value (MPO-associated MN vs. Primary MN)
Age (yr)	63.0 (56.0–68.5)	72.0 (68.0–73.0)	74.0 (64.0–74.0)	0.178	0.212
eGFR (ml/min per 1.73 m^2^)	13.9 (11.1–38.1)	21.2 (7.5–29.0)	59.9 (46.6–74.5)	0.628	0.030
eGFR after 6 mo (ml/min per 1.73 m^2^)	23.7 (19.1–55.2)	31.3 (28.5–34.7)	46.7 (37.8–47.5)	>1.000	0.432
eGFR after 12 mo (ml/min per 1.73 m^2^)	28.7 (17.6–49.6)	34.1 (30.2–36.6)	48.6 (47.3–55.5)	>1.000	0.149
U-Pro (g/gCr)	3.0 (2.5–5.4)	1.4 (0.5–1.8)	6.8 (6.1–16.7)	0.051	0.048
U-Pro after 6 mo (g/gCr)	0.8 (0.8–2.0)	0.3 (0.2–0.7)	1.3 (1.0–1.4)	0.101	0.755
U-Pro after 12 mo (g/gCr)	1.3 (0.5–1.5)	0.2 (0.1–0.4)	1.0 (0.6–1.8)	0.035	0.755
MPO-ANCA (U/ml)	25.6 (20.6–72.4)	154.0 (91.1–307.5)	N/A	0.035	N/A
Crescents (%)	54.3 (43.6–59.2)	38.5 (13.0–57.6)	N/A	0.731	N/A
Cellular and fibrocellular crescents (%)	36.4 (24.3–39.6)	39.8 (38–57.7)	N/A	0.295	N/A
Fibrous crescents (%)	15.4 (12.2–21.4)	0.0 (0.0–1.8)	N/A	0.0012	N/A

ANCA, antineutrophil cytoplasmic antibody; eGFR, estimated glomerular filtration rate; MN, membranous nephropathy; MPO, myeloperoxidase; MPO-ANCA GN, MPO-ANCA-associated glomerulonephritis; N/A, not applicable; U-Pro, urinary protein-to-creatinine ratio;. Values are presented as median (interquartile range).

## Discussion

This study aimed to prove that some cases of MPO-ANCA GN, which is accompanied by MN, may be related to MN and MPO-ANCA GN rather than each disease existing independently. In addition, the study described the clinicopathological features of MPO-ANCA GN with MN. This disease is a type of secondary MN caused by the accumulation of MPO and IgG, which is defined as MPO-associated MN. In this study, we identified MPO in 7 MPO-ANCA GN with MN cases using LC-MS/MS, which has not been previously reported and is a novel finding. In these cases, IHC and IF staining were positive for MPO along the GBM, consistent with the LC-MS/MS results. In addition, confocal IF microscopy was performed to show MPO and IgG colocalization along the GBM. Therefore, these cases are considered MPO-associated MN, similar to how new antigens for primary MN were reported.^9^ The clinical features of MPO-associated MN are massive proteinuria and severe kidney damage, whereas its pathological features include crescentic GN with subepithelial EDD accompanied by fibrous crescents and mesangial or endocapillary proliferation. Patients with MPO-associated MN had more severe proteinuria, a higher fibrous crescent rate, and a lower MPO-ANCA titer than those with MPO-ANCA GN. Meanwhile, patients with MPO-associated MN had a lower eGFR and less severe proteinuria than those with primary MN.

MN is a glomerular disease characterized by the presence of subepithelial immune deposits along the GBM, resulting in increased permeability to proteins and causing nephrotic syndrome. Immune deposits comprise antigens and antibodies that directly target the GBM. The discovery of MN-related target antigens using LC-MS/MS has been reported, and MN is defined as a type of disease based on antigens.^10^ Although ANCA GN is characterized by pauci-immune crescentic GN,^1^ ANCA GN with MN cases are rare.^2^^,^^4^^,^^11^ Particularly, several MPO-ANCA GN with MN cases exist,^5^, ^6^, ^7^^,^^12^, ^13^, ^14^, ^15^, ^16^, ^17^ some of which are positive for MPO and IgG along the GBM.^5^^,^^18^^,^^19^ Hanamura *et al.*^8^ performed immunoelectron microscopy using anti-MPO antibodies in 6 cases of ANCA GN with MN lesions to investigate the antigens within the EDD, which revealed the colocalization of MPO and glomerular immune deposits.

In our study, we performed LC-MS/MS, IHC, and IF staining to identify the target antigens in MPO-ANCA GN with MN cases. We detected moderate spectral counts of MPO in all MPO-ANCA GN with MN cases using LC-MS/MS. IHC and IF staining also confirmed the LC-MS/MS results and revealed bright granular capillary MPO along the GBM. In addition, confocal microscopy showed MPO and IgG colocalization along the GBM. These findings suggest that MPO is the target antigen for IgG, which is probably a circulating antibody to MPO, known as MPO-ANCA. The accumulation of MPO and IgG along the GBM might be related to MPO-associated MN. However, we identified MPO in 2 of the 6 MPO-ANCA GN cases using LC-MS/MS, and both IHC and IF staining for MPO were negative along the GBM in both cases. This could be because intraglomerular neutrophil infiltration might be correlated with proteomic spectral counts for MPO and because all MPO-ANCA GN and primary MN cases were negative for capillary MPO deposition.

The clinical features of MPO-associated MN showed nephrotic-range proteinuria and severe renal dysfunction. Patients with MPO-associated MN had a lower MPO-ANCA titer than those with MPO-ANCA GN. Interestingly, 2 MPO-associated MN cases showed long lasting positivity for MPO-ANCA over 10 years. The treatment strategy between MPO-associated MN and MPO-ANCA GN cases differed slightly. However, no significant difference was observed in eGFR between the 2 groups after 12 months of renal biopsy; however, significantly higher proteinuria persisted in MPO-associated MN. This is probably because MPO-associated MN has 2 components of MPO-ANCA GN and MN.

Furthermore, the pathological features of MPO-associated MN confirmed crescentic GN with subepithelial EDD accompanied by fibrous crescents. IF staining revealed fine granular IgG and C3 in all patients with MPO-associated MN, although some were slightly positive for IgA, C1q, and IgM. The rate of fibrous crescents in MPO-associated MN cases was higher than that in MPO-ANCA GN cases, indicating prolonged MPO-ANCA GN. Therefore, these results suggest that the clinicopathological features of MPO-associated MN cases are entirely different from those of MPO-ANCA GN and primary MN.

This study had some limitations, including the low incidence of MPO-associated MN cases and its retrospective single-center design. Therefore, whether MPO-associated MN is caused by preformed circulating immune complexes or immune complexes formed *in situ* that involve MPO-ANCA binding to MPO attached to the GBM cannot be revealed because no seamless renal biopsies exist at different stages of MPO-associated MN.

In conclusion, we identified MPO as the target antigen in MPO-ANCA GN with MN using LC-MS/MS, IHC, IF, and confocal microscopy. MPO-associated MN is a distinct type of MN characterized by some relationship between MPO-ANCA GN and MN. Moreover, the clinicopathological features of MPO-associated MN include proteinuria, a high fibrous crescent rate, a low MPO-ANCA titer, and severe renal dysfunction. In addition, a low MPO-ANCA titer and prolonged periods of MPO-ANCA GN might be correlated with MPO-associated MN development. Therefore, these findings provide important insights into understanding the pathogenesis of MPO-associated MN. However, further investigations are needed to reveal the mechanism of MPO-associated MN in experimental animal models.

## Disclosure

All the authors declared no competing interests.

## Supplementary Material

Supplementary File (PDF) **Figure S1.** Subepithelial electron-dense deposits in all myeloperoxidase-associated membranous nephropathy cases. **Table S1.** MPO-ANCA titers before renal biopsy in MPO-associated MN cases.

## Footnotes

Supplementary File (PDF)
**Figure S1.** Subepithelial electron-dense deposits in all myeloperoxidase-associated membranous nephropathy cases. **Table S1.** MPO-ANCA titers before renal biopsy in MPO-associated MN cases.

## References

[1] Jennette J.C. (2003). Rapidly progressive crescentic glomerulonephritis. Kidney Int.

[2] Haas M., Eustace J.A. (2004). Immune complex deposits in ANCA-associated crescentic glomerulonephritis: a study of 126 cases. Kidney Int.

[3] Tse W.Y., Howie A.J., Adu D. (1997). Association of vasculitic glomerulonephritis with membranous nephropathy: a report of 10 cases. Nephrol Dial Transplant.

[4] Nasr S.H., Said S.M., Valeri A.M. (2009). Membranous glomerulonephritis with ANCA-associated necrotizing and crescentic glomerulonephritis. Clin J Am Soc Nephrol.

[5] Manabe S., Hatano M., Nakano M., Fujii T., Nitta K., Nagata M. (2017). Myeloperoxidase-antineutrophil cytoplasmic antibody causes different renal diseases by immune-complex formation and pauci-immune mechanism: a case report. Pathol Int.

[6] Matsumoto K., Honda H., Shibata T. (2009). MPO-ANCA crescentic glomerulonephritis complicated by membranous nephropathy: MPO demonstrated in epimembranous deposits. NDT Plus.

[7] Tominaga K., Uchida T., Imakiire T. (2018). Anti-neutrophil cytoplasmic antibody-associated glomerulonephritis with detection of myeloperoxidase and phospholipase A_2_ receptor in membranous nephropathy-lesions: report of 2 patients with microscopic polyangiitis. BMC Nephrol.

[8] Hanamura K., Tojo A., Kinugasa S. (2011). Detection of myeloperoxidase in membranous nephropathy-like deposits in patients with anti-neutrophil cytoplasmic antibody-associated glomerulonephritis. Hum Pathol.

[9] Rojas-Rivera J.E., Ortiz A., Fervenza F.C. (2023). Novel treatments paradigms: membranous nephropathy. Kidney Int Rep.

[10] Ronco P., Debiec H. (2021). Membranous nephropathy: current understanding of various causes in light of new target antigens. Curr Opin Nephrol Hypertens.

[11] Neumann I., Regele H., Kain R., Birck R., Meisl F.T. (2003). Glomerular immune deposits are associated with increased proteinuria in patients with ANCA-associated crescentic nephritis. Nephrol Dial Transplant.

[12] Uyama S., Ohashi N., Iwakura T. (2013). A case presenting with the possible relationship between myeloperoxidase-antineutrophil cytoplasmic antibody associated glomerulonephritis and membranous changes of the glomerular basement membrane. CEN Case Rep.

[13] Hu Z.J., Niu K., Liu B., Shi Y.N. (2014). A case of membranous nephropathy and myeloperoxidase anti-neutrophil cytoplasmic antibody-associated glomerulonephritis. Exp Ther Med.

[14] Kanodia K., Vanikar A., Patel R. (2014). Membranous nephropathy with MPO-ANCA-associated crescentic GN. Nephrourol Mon.

[15] Carrara C., Emili S., Lin M., Alpers C.E. (2016). Necrotizing and crescentic glomerulonephritis with membranous nephropathy in a patient exposed to levamisole-adulterated cocaine. Clin Kidney J.

[16] Shimada M., Fujita T., Nakamura N. (2013). A case of myeloperoxidase anti-neutrophil cytoplasmic antibody (MPO-ANCA)-associated glomerulonephritis and concurrent membranous nephropathy. BMC Nephrol.

[17] Kanahara K., Yorioka N., Nakamura C. (1997). Myeloperoxidase-antineutrophil cytoplasmic antibody-associated glomerulonephritis with membranous nephropathy in remission. Intern Med.

[18] Kawashima S., Arimura Y., Sano K. (2013). Immunopathologic co-localization of MPO, IgG, and C3 in glomeruli in human MPO-ANCA-associated glomerulonephritis. Clin Nephrol.

[19] Hirose O., Itabashi M., Takei T., Honda K., Nitta K. (2017). Antineutrophil cytoplasmic antibody-associated glomerulonephritis with immunoglobulin deposition. Clin Exp Nephrol.

