# Supplementary material for: Myeloperoxidase-Associated Membranous Nephropathy in Antineutrophil Cytoplasmic Antibody-Associated Glomerulonephritis
Source: Kidney Int Rep. 2024 Apr 23;9(7):2240–9. doi: 10.1016/j.ekir.2024.04.041 (PMC11284429; doi:10.1016/j.ekir.2024.04.041)
Supplement: Supplementary File (PDF) — Figure S1. Subepithelial electron-dense deposits in all myeloperoxidase-associated membranous nephropathy cases. Table S1. MPO-ANCA titers before renal biopsy in MPO-associated MN cases. [file mmc1.pdf]

Supplementary Figure S1. Subepithelial electron-dense deposits in all myeloperoxidase-associated membranous nephropathy cases

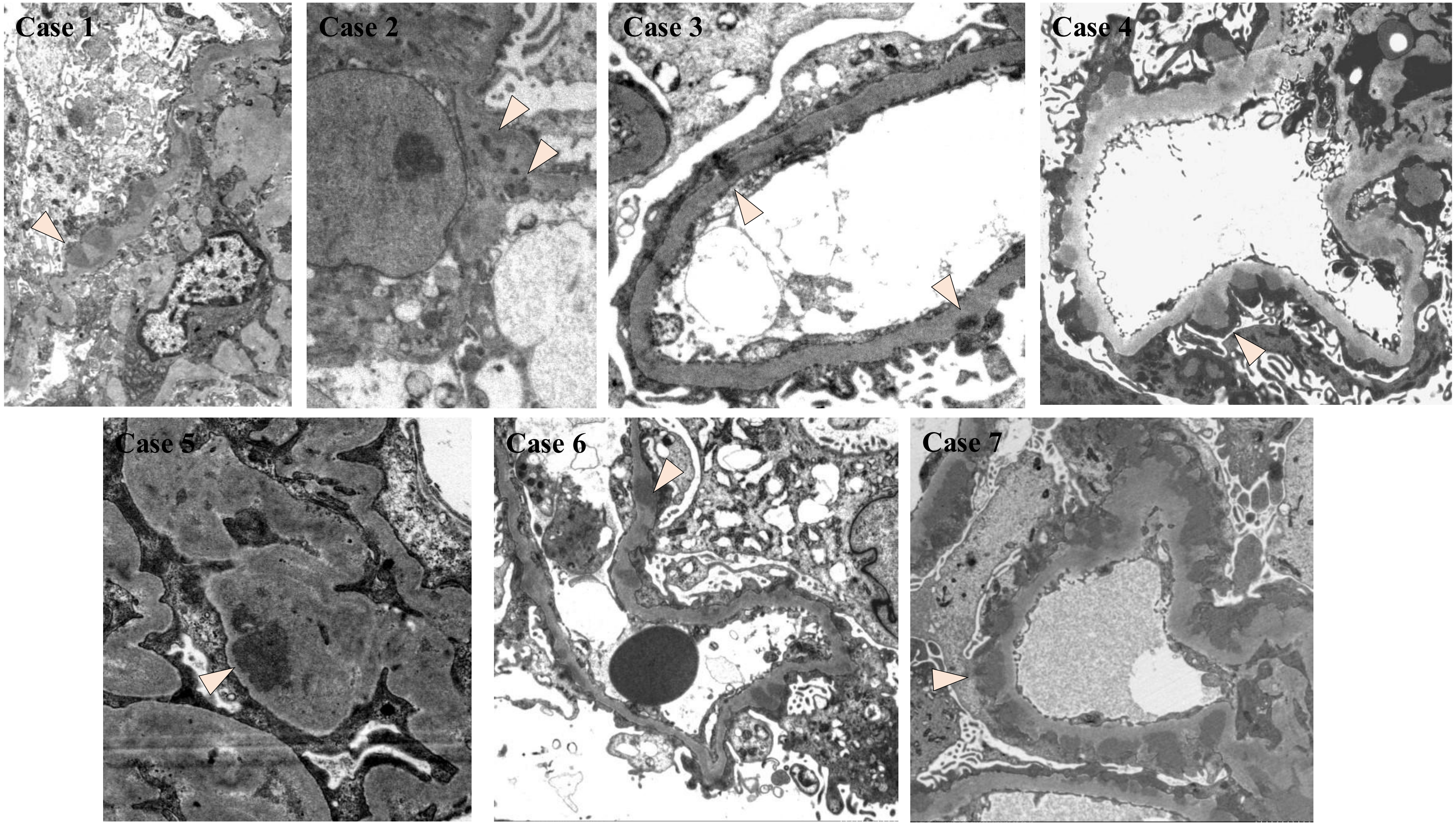

Supplementary Table S1. MPO-ANCA titers before renal biopsy in MPO-associated MN cases

	X-11 years	X-10 years	X-5 years	X-4 years	X-3 years	X-1 year	X
Case 1 (MPO-ANCA)		37 EU/mL	77 EU/mL	49 U/mL	15.7 U/mL		25.6 U/mL
Case 7 (MPO-ANCA)	29 EU/mL					25.7 U/mL	19.4 U/mL

MPO: myeloperoxidase, ANCA: anti-neutrophil cytoplasmic antibody, MN: membranous nephropathy, X: the time of renal biopsy, MPO-ANCA cutoff value before X-4 years: 20 EU/mL, MPO-ANCA cutoff value after X-4 years: 3.5 U/mL
